# Sendeng-4 Suppressed Melanoma Growth by Induction of Autophagy and Apoptosis

**DOI:** 10.1155/2021/5519973

**Published:** 2021-08-23

**Authors:** Rina Du, Pengwei Zhao, Shikui Wu, Yaoxing Gao, Rina Wu, Minli Yang, Wanying Song, Haining Gao

**Affiliations:** ^1^Inner Mongolian International Mongolian Hospital, Wulanchabudong Street, Hohhot 010090, China; ^2^Laboratory of Microbiology and Immunology, School of Basic Medical Science, Inner Mongolia Medical University, Xinhua Street, Hohhot 010059, China; ^3^College of Pharmacy, Inner Mongolia Medical University, Jinshan Street, Hohhot 010010, China; ^4^The Affiliated Hospital of Inner Mongolia Medical University, Tongdao Street, Hohhot 010010, China; ^5^Inner Mongolia Baihanxumu Biotechnology Co. LTD, Jinshan Street, Hohhot 010010, China

## Abstract

Sendeng-4 is a traditional Chinese medicine that has been successfully applied to anti-inflammatory diseases in clinical practice. Monomers within Sendeng-4 showed promising antitumor activity against lung cancer, colon cancer, and cutaneous cancer. However, potency of Sendeng-4 in melanoma has not been explored. This study aims to explore the potential application of Sendeng-4 in melanoma treatment. In the present study, we systemically investigate the possibility of Sendeng-4 for treatment of melanoma cancer in vitro by proliferation assay, colony formation, flow cell cytometry, RNA-seq, western blot, and fluorescence-based assay. Our data demonstrated that Sendeng-4 suppresses the proliferation and colony formation capacity of melanoma cells and induces cell cycle block at G2/M phase and eventually cell death. Mechanistically, transcriptome sequencing demonstrates that the PI3K-AKT pathway was significantly inactivated upon Sendeng-4 exposure, which was confirmed by western blot showing decreased phosphorylation of AKT. In addition, decreased BCL-2 expression and increased BAX expression were observed, suggesting programmed cell death via apoptosis. Moreover, LC3-II production as well as autophagosomes formation was observed as demonstrated by western blot and immunofluorescence, indicating elevated autophagy network by Sendeng-4 stimulation. Collectively, we concluded that Sendeng-4 might be used as an anticancer drug for melanoma.

## 1. Introduction

Melanoma is one of the most malignant forms of cutaneous cancer, with an increasing incidence since 1970s [1]. According to the data from World Cancer Fund, nearly 300,000 newly diagnosed melanoma were reported in 2018, accounting for about 1.7% of all cancer types [2]. Highest risk factors for melanoma include ultraviolet radiation exposure that causes DNA damage and mutations, especially in the lighter skin population and family history of melanoma with genetic instability mutations [3–5]. The most frequently mutated genes include BRAF, RAS-family genes, NF1, hTERT, CDKN2A, PTEN, TP53, and ARID2, of which BRAF mutations accounts for around 50% of melanomas [6–8]. The other risk factors include arsenic, alcohol consumption, and infection. The 5-year survival rates for melanoma patient depends on the stage at which melanoma is diagnosed, for example, the 5-year survival rate for localized melanoma is more than 98%; however, the rate for metastatic patient is only 22.5%. Surgical excision to remove localized lesion or affected lymph nodes remains the primary option for melanoma patients [9]. Additionally, combinational strategies including targeted therapy, radiation therapy, and chemotherapy showed promising clinical outcomes, and the improvement of which was further driven by clinical usage of immune checkpoints inhibitors, especially in advanced melanoma [10–13]. Although considerable progress has been made in both diagnosis and treatment in melanoma, management of the advanced metastasis melanoma and overcome drug resistance remain a challenge, largely due to lack of understanding of the molecular mechanism for melanoma development. Therefore, there is an urgent need to identify new targets and molecular markers associated with melanoma cancer progression.

Mongolian medicine Sendeng-4 is a traditional Chinese medicine composed of four medicinal herbs, including *Xanthoceras sorbifolia*, *Toosendan fructus*, *Gardeniae fructus*, and *Chebulae fructus* [14, 15]. Sendeng-4 is traditionally used as decoction; however, capsules and other formulations have also been selected in clinical practice. The most appropriate indications for Sendeng-4 include arthritis, edema, and other diseases, mainly through clearing away heat and dampness [14, 16]. Existence of monomers with anti-inflammatory and analgesic effects including gallic acid, myricetin, quercetin, and sterols, have been confirmed, which is conducive to establish a reasonable quality evaluation system and to understand the molecular mechanism of Sendeng-4 in clinical practice [14, 15]. In addition, accumulating evidence demonstrates antitumor activity against a wide range of cancer including leukemia, colon cancer, bladder cancer, and lung cancer, for Sendeng-4 containing monomers, such as *Gardenia jasminoides*, *Quercus pilosula*, and *Toosenda neem* [15, 17, 18]. However, except for anti-inflammation and antioxidation activity, the antitumor activity of Sendeng-4 is scarcely evaluated, especially in melanoma. In the present study, we systemically studied the potential antitumor activity of Sendeng-4 against melanoma, which provided new perspective for understanding the clinical application of Sendeng-4. Our preliminary findings demonstrated that Sendeng-4 suppressed melanoma proliferation in vitro, mainly through induction of cell death by autophagy and apoptosis.

## 2. Materials and Methods

### 2.1. Chemicals and Reagents

Sendeng-4 was purchased from the Chinese National Institute (Beijing, China); antibodies for AKT (4685), phosphor-AKT (4060), ERK (4695), phosphor-ERK (4370), LC3B (ab192890), Beclin-1 (3495), *β*-actin (3700), JNK (ab179461), phosphor-JNK (ab124956), p38 (8690), phosphor-p38 (4511), BAX (89477), BCL2 (15071) were purchased from Abcam and Cell Signaling Technology. CCK-8 kit for cell proliferation detection, enhanced chemiluminescent (ECL) kit for western blot, and bicinchoninic acid (BCA) kit for protein quantitation were all purchased from Helix Biotech. Rapamycin (S1039) and 3-MA (66389) were purchased form the Selleck.

### 2.2. Cells and Cell Culture

The human melanoma cancer cell line A375 (ATCC® CRL-1619) was obtained from American Type Culture Collection (ATCC). A875, HFF-1, and HSAS3 cell lines were purchased from the National Laboratory Cell Resource (NICR). Cells were maintained in a 37°C incubator with 5% CO2, cultured in DMEM medium (Corning, USA) supplemented with 10% fetal bovine serum (FBS) (Gibco, 10099–141) and 1% penicillin-streptomycin (Gibco,15070063). Cells were passaged with trypsinization every 3 days.

### 2.3. Stable Cell Line

To construct A375 cells stably transfected with GFP-LC3 or mRFP-GFP-LC3, we firstly cloned the coding sequence into the lentiviral vector (pCDHL-puro) and then transfected the expression construct with package plasmids (psPAX2 and pMD2.G) into HEK293 cells by PEI. Lentiviruses were collected 48 hours later and were concentrated by PEG8000-based method. A375 cells were transduced with lentiviruses for 48 hours and then were treated with puromycin for 48 hours to kill the untransfected cells.

### 2.4. Cell Proliferation Assay

To confirm the proliferation activity of A375 cells upon drug treatment, cells were preplated in the culture plate of 96-well form at a concentration of 5000 cells per well. Gradient dilution (3000 *μ*g/ml, 1000 *μ*g/ml, 300 *μ*g/ml, 100 *μ*g/ml, 30 *μ*g/ml, 10 *μ*g/ml, 3 *μ*g/ml, 1 *μ*g/ml, 0.3 *μ*g/ml, 0.1 *μ*g/ml, and 0.03 *μ*g/ml) of the drug or solvent was added to the cell culture medium the next day, and the cells were incubated for 3 days. The cell culture was changed to complete medium supplemented with 10 *μ*l of CCK-8 reagents and incubated at 37 degrees for another hour. Absorbance at 450 nm was quantitated by enzyme-linked immunoassay analyzer Multiskan™ FC (Thermo), and data were analyzed by GraphPad Prism 5.0.

### 2.5. Colony Formation Assay

A375 cells (1000 cells) were cultured in a plate with a diameter of 6 cm and supplemented with solvent or Sendeng-4 of different concentrations. The cells were then cultured for another 2 weeks, and the medium was changed every 3 days, until the plated single cell has grown into clone visible to the naked eye. The clones were then fixed with paraformaldehyde and then were washed and stained with crystal violet at room temperature for 30 minutes. Number of clones on every plate were counted and analyzed.

### 2.6. Next Generation Sequencing

A375 cells treated with Sendeng-4 for 3 days were collected for total RNA isolation via Trizol-based methods as described. mRNA was purified by column hybridized with Oligo dT and then reverse transcribed into cDNA. Subsequently, cDNA samples were subjected to sonication for fragmentation and then were ligated to adaptor for amplification and sequencing.

### 2.7. Western Blot

Total protein of A375 cells was isolated by RIPA lysis buffer, and the protein concentration was confirmed by BCA-base assay. 30 *μ*g of the total protein was resuspended in 1x sample buffer containing SDS, *β*-ME, Tris-HCl, and bromophenol blue and was boiled at 100 degrees for 5 minutes and then was loaded and separated by SDS-PAGE electrophoresis. Subsequently, separated proteins were then transferred onto the PVDF membrane and then were blocked with 5% nonfat milk followed by incubation with specific antibody at 4 degrees overnight. The membranes were then blotted with the corresponding secondary antibody conjugated with HRP. Signals were collected by the ECL-based method, and the intensity was quantitated by ImageJ.

### 2.8. Mitochondrial Potential

Melanoma cells' suspension was made by trypsinization, and cells were stained with JC-1 probe at 37 degrees for about 1 hour. The stained cells were then analyzed by flow cell cytometry, and positive cells were calculated by GraphPad.

### 2.9. Statistical Analysis

All the experiments were performed in triplet, and result was expressed as mean ± SD. All the data were analyzed by GraphPad Prism 5, and *t*-test and one-way ANOVA were used to calculate the significance between treatment and groups.

## 3. Results

### 3.1. Sendeng-4 Suppressed Melanoma Cell Growth In Vitro

**Figure 1:**
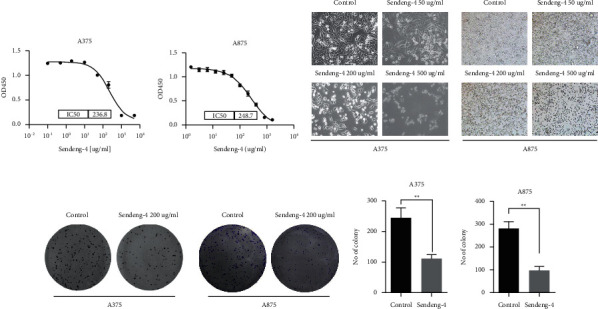
Seneng-4 suppressed proliferation and colony formation of melanoma cells. (a, b) The inhibitory curve assessed by CCK-8 assay in both A375 cells and A875 cells, *n* = 5. (c, d) Representative images of melanoma cells treated with different concentrations of Sendeng-4, 100x. (e, f) Colony formation stained with crystal violet of A375 and A875 cells upon treatment with Sendeng-4. (g, h). Quantitation of the colony formation assay, *n* = 3, ^*∗∗*^: *p* < 0.01.

Although existence of monomers with antitumor activity in Chinese compound medicine-Mongolian Sendeng-4 has been confirmed by several reports, its potential application in tumor treatment has not been explored. To investigate whether Sendeng-4 has antitumor activity in cutaneous cancer, we firstly analyzed the potency of Sendeng-4 on melanoma cell proliferation in both A375 and A875 cells. The results showed that Sendeng-4 suppressed melanoma cells in a dose-dependent manner, with an IC50 of 233.8 ug/ml for A375 and 248.7 ug/ml as shown in Figures 1(a) and 1(b). However, no significant cytotoxicity effects were observed with normal skin cells (Supplemental Figure 1A). We hypothesized that Sendeng-4 might inhibit cell growth through some unknown pathway. Interestingly, as compared to the control group, morphology of cells stimulated with higher concentration of Sendeng-4, exhibiting spherical phenotype, suggesting cell cycle arrest or cell death (Figures 1(c) and 1(d)). To extend our findings that Sendeng-4 exerted inhibitory effect on melanoma growth in vitro, we employed congenic formation assay to assess the toxicity of Sendeng-4 on A375 cells. Colony formation capacity was significantly impaired by Sendeng-4 (Figures 1(e)–1(h)). Collectively, these findings suggest that Sendeng-4 is a potential candidate drug for melanoma.

### 3.2. Sendeng-4 Induced Melanoma Cell Cycle Arrest and Cell Death

**Figure 2:**
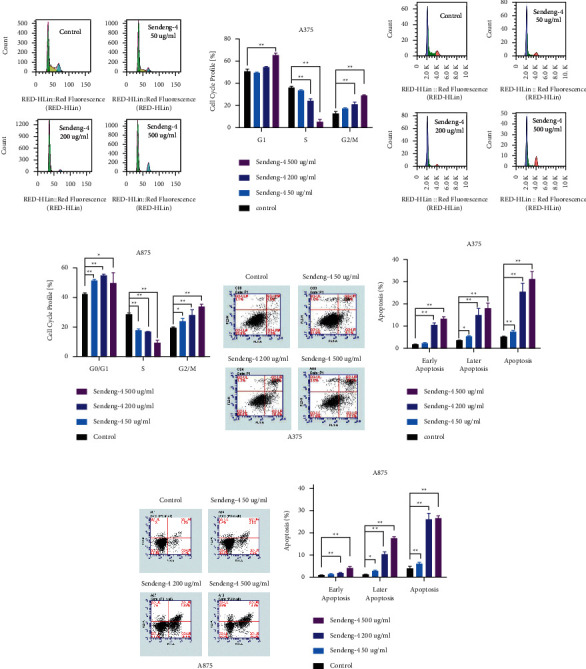
Sendeng-4 treatment induced cell cycle arrest and death in A375 cells. (a, b) Cell cycle profile of A375 cells determined by flow cell cytometry, *n* = 3. (c, d) Cell cycle profile of A875 cells determined by flow cell cytometry, *n* = 3. (e, f) Cell apoptosis detected by Annexin-V/PI staining and cell flow cytometry in A375 cells, *n* = 3. (g, h) Cell apoptosis detected by Annexin-V/PI staining and cell flow cytometry in A875 cells.

To check whether Sendeng-4 leads to cell cycle arrest, A375 and A875 cells were fixed with ice-cold methanol and then stained with propidium iodide (PI) to analyze DNA content by flow cell cytometry. As expected, the cell cycle profile of melanoma cells demonstrated significant G2/M phase block (Figures 2(a)–2(d)), consistent with the previous findings that more spherical cells were observed when exposed to higher dose Sendeng-4. Interestingly, percentage of the S-phase cells was also decreased in higher dose groups. Although it remains unclear how Sendeng-4 stimulation leads to cell cycle block, accumulating studies have reported the cell death induced by long-time cell cycle arrest. In support of our hypothesis, cell apoptosis upon treatment with Sendeng-4 was assessed by Annexin-V/PI staining and analyzed by flow cell cytometry. As observed in Figures 2(e)–2(h), both early apoptosis and late apoptosis were significantly elevated. In addition, mitochondrion membrane potential was determined by flow cell cytometry, and Sendeng-4 treatment induced potential lose in a dose-dependent manner (Supplemental Figures 1B and 1C).

### 3.3. Sendeng-4 Inhibits PI3K-AKT Pathway

**Figure 3:**
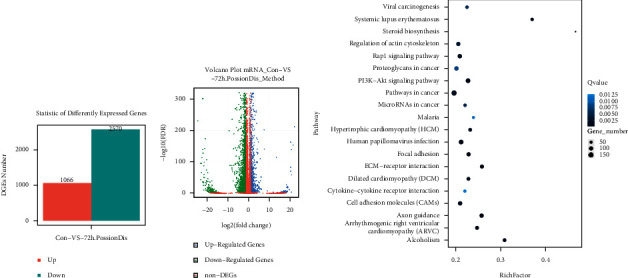
Sendeng-4 treatment leads to gene expression changes determined by RNA-seq. (a) Number of decreased and increased genes upon treatment Sendeng-4 stimulation. (b) Volcano plot of differentially expressed genes. (c) Bubble chart of KEGG pathways affected by Sendeng- treatment.

**Figure 4:**
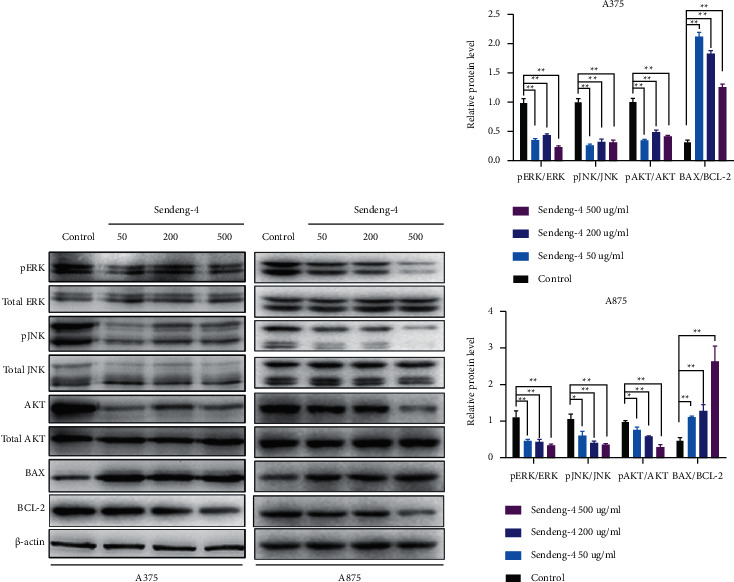
Sendeng-4 promote cell apoptosis and inhibit MAPK and PI3K/AKT signaling pathways. (a) A375 and A875 cells were treated with different concentrations of Sengdeng-4 for 12 h Western blot analysis was performed to measure protein levels of phospho-ERK (p-ERk), total ERK, phospho-JNK (p-JNK), total JNK, p-AKT, total AKT, BAX, and BCL-2 in A375 cells. Β-actin was used as an internal control. (b) Quantification of (a).

In order to further explore the potential effect for Sendeng-4 upon melanoma cells, whole cell transcriptome was performed to assess the expression profile of A375 cells. As compared with the control group, 1066 genes were upregulated and 2570 genes were downregulated in the Sendeng-4 exposure group (Figures 3(a) and 3(b)). Kyoto Encyclopedia of Genes and Genomes (KEGG) including cell adhesion molecules (CAMs) analysis demonstrated pathways in cancer, PI3K-AKT signaling pathway, focal adhesion pathways, and regulation of actin cytoskeletons (Figure 3(c)). Of note, the PI3K-AKT pathway accounts for physiological roles such as cell proliferation and migration, which are consistent with documented findings that Sendeng-4 containing monomers exert antitumor activity through MAPK pathway and cell apoptosis. Thus, we then sought to clarify whether MAPK-related pathways could be affected by Sendeng-4 stimulation. We observed that phosphorylation of AKT at serine 473 was significantly inhibited (Figures 4(a) and 4(b)), suggesting decreased activity of the AKT signaling pathways, which are correlated with cell proliferation, metastasis, and many other physiological processes. Additionally, phosphorylation of ERK and JNKs were also decreased upon Sendeng-4 stimulation, suggesting a broad impact on cell growth. Moreover, proapoptotic factor BAX was significantly induced; on the contrary, the antiapoptotic factor BCL-2 was dramatically decreased in a dose-dependent manner, which were all consistent with the phenotype determined by flow cytometry.

### 3.4. Autophagy of Melanoma Cells Were Elevated upon Sendeng-4 Stimulation

**Figure 5:**
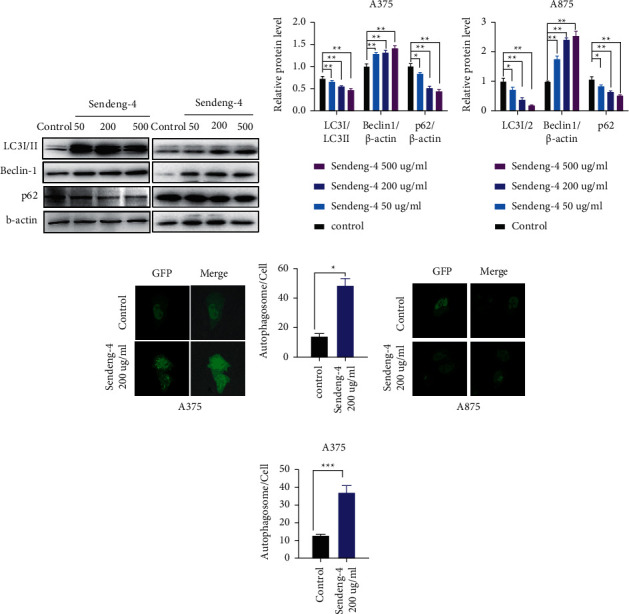
Sendeng-4 promote cell autophagy. A375 and A875 cells were treated with different concentrations of Sengdeng-4 for 12 h. (a) Western blot analysis was performed to measure protein levels of LC3I/II, beclin-1, and p62. *ß*-Actin was used as an internal control. (b, c) Quantification of (a). (d, f) Immunofluorescence was performed to measure cell autophagy. (f, g) Quantification of (d).

Autophagy arises in cells to maintain homeostasis by degradation and recycling of cellular components. Autophagy is also elevated upon stimulation with exogenous stress such as bacteria, virus, or other forms of pathogens. Defects in autophagy pathway have been reported to be related with a variety of diseases including tumor, infectious, and neurodegeneration diseases [19, 20]. Of note, autophagy is tightly regulated via phosphorylation of mTOR by the PI3K-AKT pathway [21]. Therefore, we then sought to validate the potential outcome for autophagy pathway upon Sendeng-4 stimulation. As shown in Figures 5(a)–5(c), compared with the vehicle group, Sendeng-4 treatment leads to dramatically increase of LC3 (LC3-I and LC3-II), which is a well-characterized marker for autophagy, suggesting elevation of the autophagic flux. Accordingly, expression of Beclin-1 is also increased upon Sendeng-4 treatment (Figures 5(a)–5(c)). Moreover, formation of autophagosome upon Sendeng-4 stimulation was observed by fluorescence reporter, which is consistent with the elevation of LC3 (Figures 5(d)–5(g)).

### 3.5. Sendeng-4 Treatment Increased Autophagic Flux

**Figure 6:**
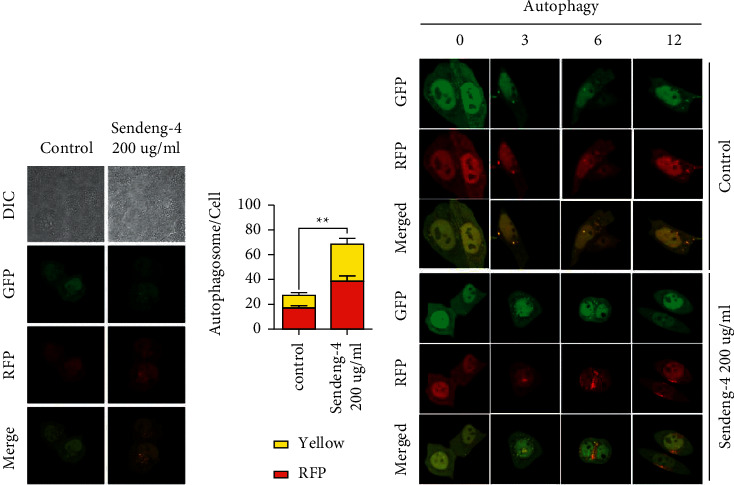
Autophagic flux in elevated upon Sendeng-4 stimulation. (a) Detection of autophagic flux with the mRFP-GFP-LC3 reporter, and confocal microscopy images merger with GFP and RFP fluorescence of representative cells. (b) Calculation of red foci and yellow foci are shown in the figure, ^*∗∗*^: *p* < 0.01. (c) Autophagic flux assessed with the mRFP-GFP-LC3 reporter at different time points upon Sendeng-4 treatment.

In order to further confirm the stimulatory effect of Sendeng-4 on autophagy, we then established melanoma cell lines that stably expressed with RFP-GFP-LC3. As compared with the control cells, more autophagosomes positive for both RFP and GFP were observed upon Sendeng-4 treatment (Figures 6(a)–6(c)). In addition, more autophagosomes that are positive for RFP staining were observed in cells treated with higher dose Sendeng-4. Taking together, these findings indicated that Sendeng-4 strongly activated the autophagic flux in melanoma cells.

Regarding the bilateral roles of autophagy in tumor development, we next checked whether the Sendeng-4 mediated autophagy activation promotes or inhibits melanoma tumor growth. To this end, melanoma cells were treated with Sendeng-4 and/or autophagy inhibitor (3-MA) or activator (rapamycin). Interestingly, inhibition of autophagy by 3-MA alleviated the Sendeng-4 mediated melanoma cell death and, on the contrary, was augmented by autophagy activation, as evidenced by the half maximal inhibition concentration of Sendeng-4 on melanoma cells (Supplemental Figures 1D and 1E). Furthermore, the antitumor roles of autophagy under the context of Sendeng-4 treatment was further demonstrated by upregulation of BAX and downregulation of BCL2 as determined by western blot (Supplemental Figures 2A–2C). Collectively, Sendeng-4 suppressed melanoma activity via activation of autophagy.

## 4. Discussion

Autophagy is an evolutionarily conserved pathway regulating the cell hemostasis through degradation and recycling under both physiological and pathological conditions [21, 22]. Three types of autophagy have been described such as macroautophagy, microautophagy, and chaperone-mediated autophagy. Macroautophagy is mainly responsible for bulk degradation of the cytoplasmic components upon stressful conditions, which involves formation of bilayer spherical autophagosomes mediated by ATGs [21, 22]. For example, upon stimulation, such as starvation or growth factor deprivation, the upstream suppressor mTOR was inactivated by phosphorylation, thus leading to ULK1/2 activation and phosphorylation of downstream effectors [23]. Notably, posttranslational modification of ATG8 with phosphatidylethanolamine (PE) is a crucial event for the formation of autophagosomes [24]. The resultant vesicle was then fused with lysosome for subsequent degradation and recycling [25]. The microautophagy differs from macroautophagy by direct engulfment of the cytosolic material through inward folding of the lysosomal membrane. The chaperone-mediated autophagy is specific to mammalian cells, referring to lysosome-dependent degradation of HSP70 complex chaperoned molecules, such as nonessential or wrong folding proteins [25–28].

As documented, roles of autophagy in cancer development and progression remain controversial. Autophagy is required for the hemostasis of cell to prevent tumorigenesis, and absence of autophagy master genes, such as Beclin-1, ATG5, and UVRAG, has been reported in multiple cancers including breast cancer lung cancer [29, 30]. On the contrary, autophagy is believed to promote tumorigenesis by antagonizing genotoxic stress and inflammation and microenvironment remodeling. In accordance with this notion, advanced form of cancers always exhibits autophagic-proficient state [31, 32]. Interestingly, cancers with Ras mutation are highly dependent on autophagy. As demonstrated in a *Drosophila melanogaster* malignant tumor model, tumors cells with Ras mutation induced higher levels of autophagy at both tumor cells and surrounding normal cells, thus providing nutrients to facilitate tumor growth [33]. Accordingly, autophagy restraining by pharmacological inhibitors provided new strategies for cancer treatment [34]. In addition, specific gut microbes hijacked the autophagy network to alter colorectal cancer chemotherapeutic response, thus leading to chemoresistance and recurrence [35]. Collectively, roles of autophagy in tumorigenesis and development are largely context-dependent.

Cutaneous melanoma ranks the most aggressive form of cancer, especially the cases with distal metastasis. While new approvals have yet been granted in the past decades, 5-year survival for metastatic melanoma remains less than 30% [1]. Aberrant expression autophagic genes have been documented as observed in clinical samples, and LC3-II expression and beclin-1 in melanoma patient parallels disease stage and progression. Additionally, clinical administration of temozolamide and sorafenib led to elevated autophagic activity, which warranted benefits from chemotherapy [36]. In accordance with this, combinational therapy of chemotherapy regimens and autophagic inhibitors is now been tested in numerous clinical trials [37].

In the present study, for the first time that we demonstrated the potential application of the traditional Chinese medicine Sendeng-4 in cutaneous melanoma treatment. In brief, Sendeng-4 stimulation leads to delayed proliferation, impaired colony formation, cell cycle arrest, and eventually cell death. Mechanically, Sendeng-4 treatment inactivated the PI3K-AKT pathway, as indicated by the decreased phosphorylation of AKT. Furthermore, the autophagic activity was dramatically increased upon Sendeng-4 treatment in melanoma cells. As documented, Sendeng-4 has been administrated to alleviate inflammation and pain in clinical practice for long time [14,15]. However, inflammation is tightly correlated with tumor development contributing to its growth and metastasis by remodeling the tumor microenvironment. Moreover, chronic inflammation increased the risk for tumorigenesis, and anti-inflammation agents have also been administrated to cancer patients, mostly in a combinational regimen. As a traditional Chinese medicine extracted form *Xanthoceras sorbifolia*, *Toosendan fructus*, *Gardeniae fructus*, and *Chebulae fructus*, monomers isolated from these herbs have been documented with roles in antitumor effect. For example, recently study reported that polymethoxyflavones from *Gardenia oudiepe* (Rubiaceae) induce cytoskeleton disruption-mediated apoptosis and sensitize BRAF-mutated melanoma cells to chemotherapy [38]. In addition, antitumor effects have also been reported by other group that the ethanol extract of baked *Gardeniae fructus* exhibits in vitro and in vivo antimetastatic and antiangiogenic activities in malignant cancer cells by suppression of the NF-*κ*B and HIF-1*α* pathways [39]. Consistently, our present study suggested similar clues for clinical application of Sendeng-4 in cancer treatment, whereas with a new molecular mechanism. However, further research should be undertaken to investigate the whether this antitumor was due to polymethoxyflavones or other kinds of monomers. Collectively, our data demonstrated possibility to treat melanoma with Sendeng-4, which is a traditional anti-inflammation Chinese medicine.

## 5. Conclusions

In conclusion, our findings demonstrated that Sendeng-4 might be used as an anticancer drug for melanoma. Mechanically, the underlying molecular interplay between PI3K-AKT pathway and autophagy network might be responsible for Sendeng-4 induced cell suppression and death. Nevertheless, further studies should be warranted to fully elucidate the physiological process affect by Sendeng-4 and to design the most suitable therapy schedule. We hope that our findings could provide a new insight for treatment of melanoma cancer.

## Data Availability

All the data in this manuscript are available upon request.

## Abbreviations

ATCC: American Type Culture Collection
CAMs: Cell adhesion molecules
KEGG: Kyoto Encyclopedia of Genes and Genomes
PI: Propidium iodide
PE: Phosphatidylethanolamine.

## Supplementary Materials

Supplementary Materials *Supplemental Figure 1.* (A) Cell viability assay determined by CCK-8 assay in melanoma cell lines (A375 and A875) and normal skin cell lines (HFF-1 and HSAS3). (B, C) Mitochondrion membrane potential analyzed by JC-1 staining and flow cell cytometry. (D, E) Cell viability assay determined by CCK-8 assay in melanoma cell lines (A375 and A875) upon combined treatment of Sendeng-4 with autophagy modulators. *Supplemental Figure 2.* (A, B) Apoptosis of A375 cells treated with Sendeng-4 or/and autophagy modulator (3-MA: inhibitor; rapamycin: activator) analyzed by flow cell cytometry. (C) Western blot analysis of apoptosis-related markers in A375 melanoma cells upon treatment with Sendeng-4 or/and autophagy modulator (3-MA: inhibitor; rapamycin: activator).

## References

[1] Schadendorf D., Van Akkooi A. C. J., Berking C. (2018). Melanoma. *The Lancet*.

[2] Ortega M., Fraile‑Martínez O., García‑Honduvilla N. (2020). Update on uveal melanoma: translational research from biology to clinical practice (Review). *International Journal of Oncology*.

[3] Gandini S., Autier P., Boniol M. (2011). Reviews on sun exposure and artificial light and melanoma. *Progress in Biophysics and Molecular Biology*.

[4] Boniol M., Autier P., Boyle P., Gandini S. (2012). Cutaneous melanoma attributable to sunbed use: systematic review and meta-analysis. *British Medical Journal*.

[5] Berwick M., Erdei E., Hay J. (2009). Melanoma epidemiology and public health. *Dermatologic Clinics*.

[6] Noonan F. P., Zaidi M. R., Wolnicka-Glubisz A. (2012). Melanoma induction by ultraviolet A but not ultraviolet B radiation requires melanin pigment. *Nature Communications*.

[7] Shain A. H., Yeh I., Kovalyshyn I. (2015). The genetic evolution of melanoma from precursor lesions. *New England Journal of Medicine*.

[8] Cancer Genome Atlas N. (2015). Genomic classification of cutaneous melanoma. *Cell*.

[9] Miller K. D., Fidler-Benaoudia M., Keegan T. H. (2020). Cancer statistics for adolescents and young adults. *CA: A Cancer Journal for Clinicians*.

[10] Ives N. J., Suciu S., Eggermont A. M. M. (2017). Adjuvant interferon-*α* for the treatment of high-risk melanoma: an individual patient data meta-analysis. *European Journal of Cancer*.

[11] Larkin J., Ascierto P. A., Dréno B. (2014). Combined vemurafenib and cobimetinib in BRAF-mutated melanoma. *New England Journal of Medicine*.

[12] White P. S., Pudusseri A., Lee S. L., Eton O. (2017). Intermittent dosing of dabrafenib and trametinib in metastatic BRAFV600E mutated papillary thyroid cancer: two case reports. *Thyroid*.

[13] Robert C., Karaszewska B., Schachter J. (2015). Improved overall survival in melanoma with combined dabrafenib and trametinib. *New England Journal of Medicine*.

[14] Wang X., Li D., Jiang M. (2018). Nearest neighbor algorithm coupled with metabonomics to study the therapeutic mechanism of sendeng-4 in adjuvant-induced rheumatoid arthritis rat. *Evid Based Complement Alternat Med*.

[15] Zi T., Yu D. (2015). A network pharmacology study of Sendeng-4, a Mongolian medicine. *Chinese Journal of Natural Medicines*.

[16] Zhang Y., Du R., Zhao P. (2020). Preparation and characterization of natural quercetin-based Mongolia Medicine SenDeng-4 nanoemulsion (N-QUE-NE) and its antibacterial activity. *Current Drug Delivery*.

[17] Hu L., Zhao J., Liu Y. (2020). Geniposide inhibits proliferation and induces apoptosis of diffuse large B-cell lymphoma cells by inactivating the HCP5/miR-27b-3p/MET axis. *International Journal of Medical Sciences*.

[18] Xia W., Khan I., Li X.-A. (2020). Adaptogenic flower buds exert cancer preventive effects by enhancing the SCFA-producers, strengthening the epithelial tight junction complex and immune responses. *Pharmacological Research*.

[19] Djajadikerta A., Keshri S., Pavel M., Prestil R., Ryan L., Rubinsztein D. C. (2020). Autophagy induction as a therapeutic strategy for neurodegenerative diseases. *Journal of Molecular Biology*.

[20] Levy J. M. M., Towers C. G., Thorburn A. (2017). Targeting autophagy in cancer. *Nature Reviews Cancer*.

[21] Xie Z., Klionsky D. J. (2007). Autophagosome formation: core machinery and adaptations. *Nature Cell Biology*.

[22] Mizushima N., Yoshimori T., Ohsumi Y. (2011). The role of atg proteins in autophagosome formation. *Annual Review of Cell and Developmental Biology*.

[23] Russell R. C., Tian Y., Yuan H. (2013). ULK1 induces autophagy by phosphorylating Beclin-1 and activating VPS34 lipid kinase. *Nature Cell Biology*.

[24] Kabeya Y., Mizushima N., Yamamoto A. (2004). LC3, GABARAP and GATE16 localize to autophagosomal membrane depending on form-II formation. *Journal of Cell Science*.

[25] Fujita N., Hayashi-Nishino M., Fukumoto H. (2008). An Atg4B mutant hampers the lipidation of LC3 paralogues and causes defects in autophagosome closure. *Molecular Biology of the Cell*.

[26] Park S., Choi S.-G., Yoo S.-M., Son J. H., Jung Y.-K. (2014). Choline dehydrogenase interacts with SQSTM1/p62 to recruit LC3 and stimulate mitophagy. *Autophagy*.

[27] Fader C. M., Sánchez D. G., Mestre M. B., Colombo M. I. (2009). TI-VAMP/VAMP7 and VAMP3/cellubrevin: two v-SNARE proteins involved in specific steps of the autophagy/multivesicular body pathways. *Biochimica et Biophysica Acta (BBA) - Molecular Cell Research*.

[28] Furuta N., Fujita N., Noda T., Yoshimori T., Amano A. (2010). Combinational soluble N-Ethylmaleimide-sensitive factor Attachment protein receptor proteins VAMP8 and Vti1b mediate fusion of antimicrobial and canonical autophagosomes with lysosomes. *Molecular Biology of the Cell*.

[29] Qu X., Yu J., Bhagat G. (2003). Promotion of tumorigenesis by heterozygous disruption of the beclin 1 autophagy gene. *Journal of Clinical Investigation*.

[30] Liang C., Feng P., Ku B. (2006). Autophagic and tumour suppressor activity of a novel Beclin1-binding protein UVRAG. *Nature Cell Biology*.

[31] Yue Z., Jin S., Yang C., Levine A. J., Heintz N. (2003). Beclin 1, an autophagy gene essential for early embryonic development, is a haploinsufficient tumor suppressor. *Proceedings of the National Academy of Sciences*.

[32] Kang R., Zeh H. J., Lotze M. T., Tang D. (2011). The Beclin 1 network regulates autophagy and apoptosis. *Cell Death & Differentiation*.

[33] Katheder N. S., Khezri R., O’Farrell F. (2017). Microenvironmental autophagy promotes tumour growth. *Nature*.

[34] White E., DiPaola R. S. (2009). The double-edged sword of autophagy modulation in cancer. *Clinical Cancer Research*.

[35] Yu T., Guo F., Yu Y. (2017). Fusobacterium nucleatum promotes chemoresistance to colorectal cancer by modulating autophagy. *Cell*.

[36] Hayes A. J., Maynard L., Coombes G. (2016). Wide versus narrow excision margins for high-risk, primary cutaneous melanomas: long-term follow-up of survival in a randomised trial. *The Lancet Oncology*.

[37] Swampillai A. L., Salomoni P., Short S. C. (2012). The role of autophagy in clinical practice. *Clinical Oncology*.

[38] Gonçalves de Oliveira-Júnior R., Marcoult-Fréville N., Prunier G. (2020). Polymethoxyflavones from Gardenia oudiepe (Rubiaceae) induce cytoskeleton disruption-mediated apoptosis and sensitize BRAF-mutated melanoma cells to chemotherapy. *Chemico-Biological Interactions*.

[39] Im M., Kim A., Ma J. Y. (2016). Ethanol extract of baked Gardeniae Fructus exhibits in vitro and in vivo anti-metastatic and anti-angiogenic activities in malignant cancer cells: role of suppression of the NF-*κ*B and HIF-1*α* pathways. *International Journal of Oncology*.

