# Supplementary figures and images for: Sendeng-4 Suppressed Melanoma Growth by Induction of Autophagy and Apoptosis
Source: Evid Based Complement Alternat Med. 2021 Aug 23;2021:5519973. doi: 10.1155/2021/5519973 (PMC8407990; doi:10.1155/2021/5519973)

Supplemental Figure 1

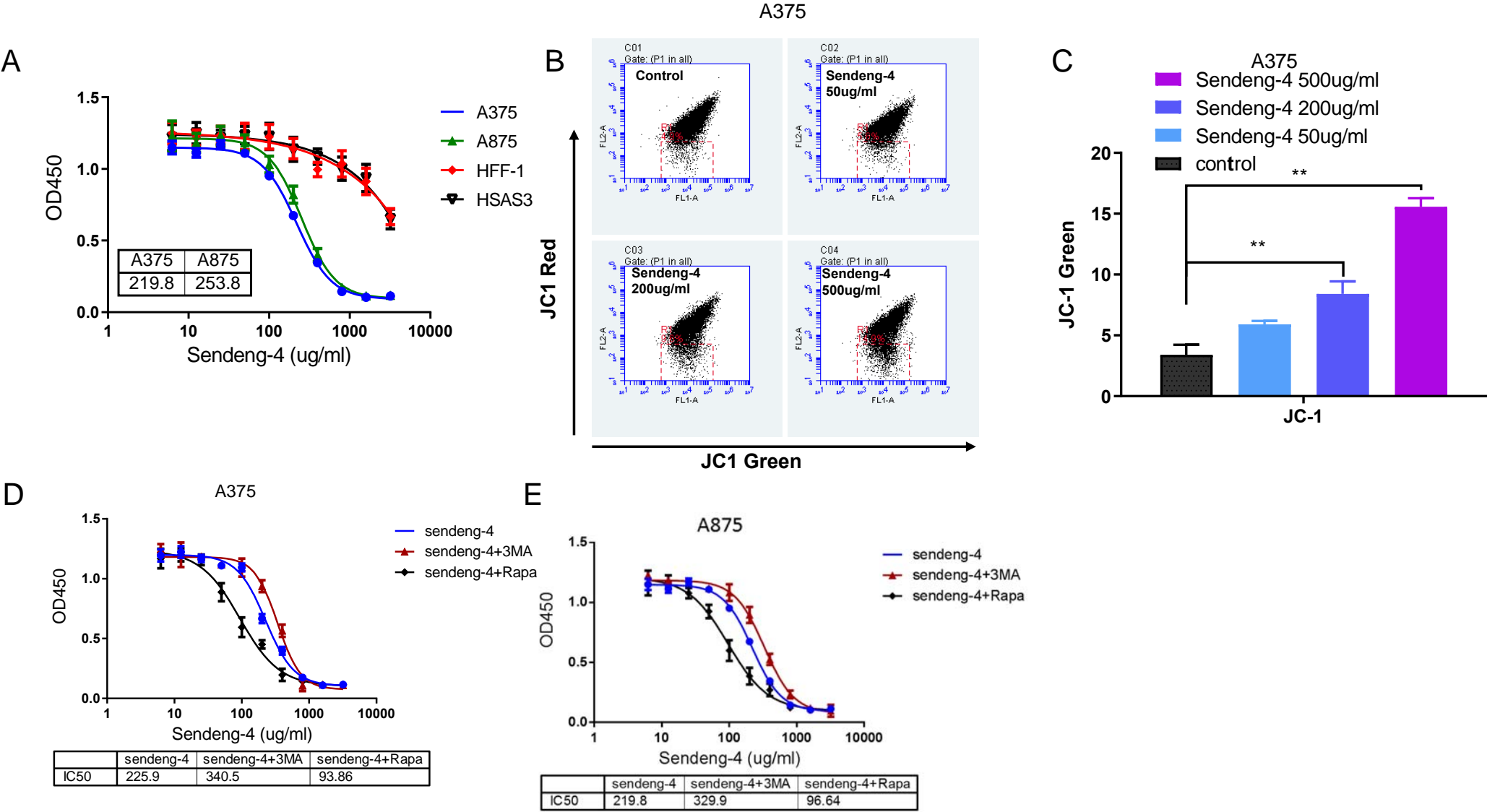

Supplemental Figure 2

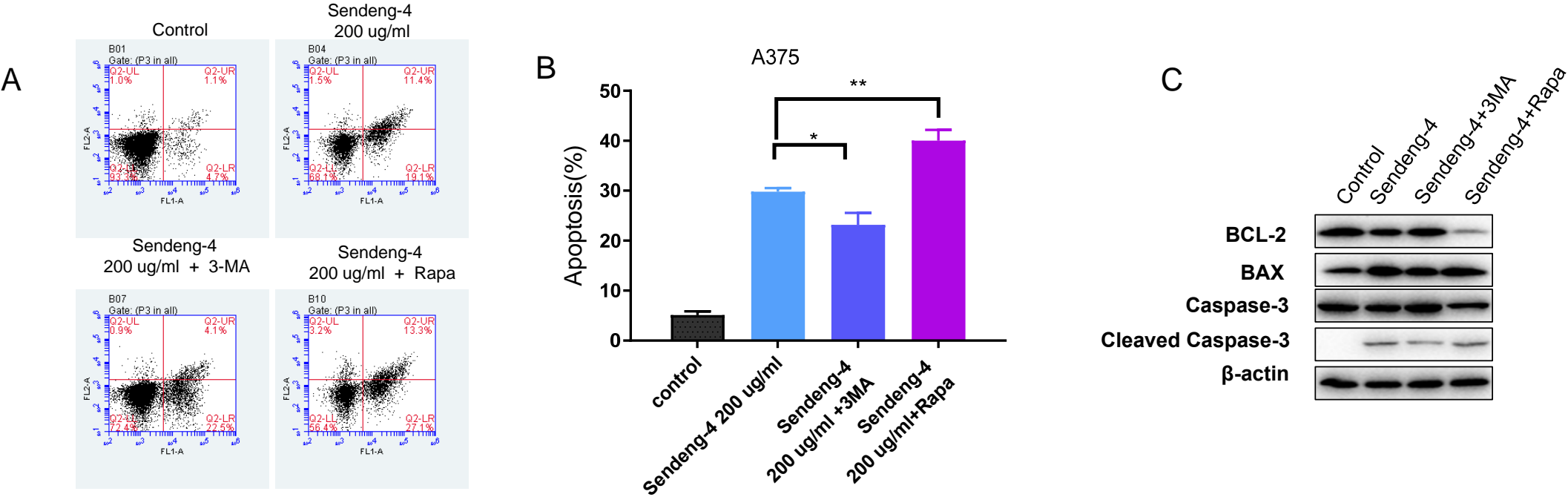

Supplement: Supplementary Materials — Supplemental Figure 1. (A) Cell viability assay determined by CCK-8 assay in melanoma cell lines (A375 and A875) and normal skin cell lines (HFF-1 and HSAS3). (B, C) Mitochondrion membrane potential analyzed by JC-1 staining and flow cell cytometry. (D, E) Cell viability assay determined by CCK-8 assay in melanoma cell lines (A375 and A875) upon combined treatment of Sendeng-4 with autophagy modulators. Supplemental Figure 2. (A, B) Apoptosis of A375 cells treated with Sendeng-4 or/and autophagy modulator (3-MA: inhibitor; rapamycin: activator) analyzed by flow cell cytometry. (C) Western blot analysis of apoptosis-related markers in A375 melanoma cells upon treatment with Sendeng-4 or/and autophagy modulator (3-MA: inhibitor; rapamycin: activator). [file 5519973.f1.pdf]
